# Cutaneous Malignant Melanoma in Central Iran: A 20-Year Study

**DOI:** 10.5812/ircmj.5364

**Published:** 2013-08-05

**Authors:** Mohammad Taghi Noorbala, Saman Mohammadi, Mohammad Noorbala

**Affiliations:** 1 Dermatology Department, Shahid Sadoughi University of Medical Sciences, Yazd, IR Iran

**Keywords:** Cutaneous Malignant Melanoma, Epidemiology, Prevalence, Survival Rate

## Abstract

**Background:**

Skin cancers are the most common cancers around the world. Cutaneous malignant melanoma (CMM) is the malignancy of melanocytes that are mainly located in the skin and mucous membranes.

**Objective:**

This study tried to evaluate the incidence and mean survival time of cutaneous malignant melanoma (CMM) in Yazd, Iran. It seems that the epidemiology and clinical aspects of CMM in Iran are different from those in other parts of the world; also due to the limited and scattered studies there isn't lot in the literature regarding CMM in Iran.

**Materials and Methods:**

This study used data obtained from the cancer registry center in the province of Yazd for a period of 21 years (1988 – 2008). Population and statistical data were gathered from “National Organization for Civil Registration”. Population-based data were analyzed, focusing on the incidence and mean survival time over this 21 year period.

**Results:**

The mean incidence rate for CMM in Yazd-Iran between 1988 and 2008 was 0.40 per 100,000 for males and 0.27 per 100,000 for females per year, and the incidence of CMM was relatively constant during this period of time. The mean survival rates for women were better than men (80.5% and 76.3% respectively).

**Conclusions:**

CMM in Yazd is a low-incidence skin tumor that shows a relatively fixed incidence between 1988 and 2008, Higher incidences of CMM were found in sun-exposed areas (especially head and neck areas), with more incidence in men. Skin cancers and CMM incidence in Iran is lower than western countries, most probably due to geographical zone, genetic factors, skin type, society-related customs including clothing styles.

## 1. Background

Skin cancers are the most common cancers around the world (1-5). Cutaneous malignant melanoma (CMM) is the malignancy of melanocytes that are mainly located in the skin and mucous membranes. As CMM has the highest rate of skin cancer–related deaths worldwide, its early detection is the mainstay of reducing mortality. Melanoma shows great potential for dissemination, and for this reason it constitutes one of the severe tumors amongst skin lesions, with high mortality rates when diagnosed late (6-8). Solar UV-Rays are amongst the most important causes of skin cancers (including CMM). In tropical countries with sunny climates, working outdoor increases the risk of skin malignancy in fair skin people (1, 5, 9). The frequency of such lesions increases with age (10).

Amongst other causes of skin cancer are exposure to X-Ray, viruses and diseases that weaken the immune system (1, 2, 6, 11-14). Nevi are generally benign and very scarcely transform to skin cancers (especially CMM), and in such a case congenital nevi pose the highest and common moles with the lowest risk of transformation (1-15%) (1-3, 15). Skin malformations are easily visible even with untrained-bare eye. Most skin cancers can be easily diagnosed and efficiently treated if patients are provided with basic information, and medical students are properly trained (5, 9). In Iran higher incidences of skin cancers and CMM can be expected because of direct bright sunlight in most seasons of the year, direct exposure of individuals to solar UV-Rays, and insufficient use of protective measures such as apparel and hats. Studies of skin cancer situation in Iran have been limited and scattered (16, 17).

## 2. Materials and Methods

This is a cross-sectional analytic study using simple census sampling. The sample population consists of patients referred to public health centers in province of Yazd with the diagnosis of skin cancer confirmed by pathology between 1988 and 2008. We gathered descriptive data from cancer registry center in the province, and death records and causes for deaths from patient's profiles at times of admission and discharge. Population and statistical data were gathered from “National Organization for Civil Registration”. For analysis the data we used chi-square test to calculate prevalence of Melanoma, Kaplan – Meier and Mantel-Cox test to calculate annual death rates and mean five year survival for CMM.

## 3. Results

In a period of 21 years (1988 – 2008), 15056 cases of cancers were recorded; 3071 cases had Non-Melanoma skin cancer (NMSC) and 71 cases had CMM. Table 1 demonstrates the prevalence of these cancers categorized by gender and registration year. The prevalence of NMSC during this period was 18 – 22.4% with mean of 20.39% of all body cancers. A total of 71 cases of CMM were reported; 28 (39.43%) women and 43 (60.56%) men. This was about 2.3% of all recorded cases with skin cancer. The frequencies of CMM based on age groups are shown in table 2. More than 42% of patients were between 40 – 60 years of age. 

**Table 1.** Distribution of Reported Cancers (All, NMSC, CMM) in Different Years (1988-2008)

Year	Malignant Melanoma			Non-Melanoma Skin Cancer	All Reported Cancers
	Total	Male	Female		
**1988**	4	3	1	153 (23.3%)	656
**1989**	5	2	3	162 (22.4%)	723
**1990**	2	2	0	148 (21.5%)	687
**1991**	1	0	1	137 (19.6%)	698
**1992**	3	2	1	149 (20.9%)	712
**1993**	4	2	2	136 (21.8%)	623
**1994**	3	2	1	129 (19.5%)	659
**1995**	5	2	3	134 (18.5%)	724
**1996**	4	3	1	117 (18.6%)	628
**1997**	3	2	1	146 (19%)	767
**1998**	3	3	0	143 (18.8%)	785
**1999**	4	2	2	142 (19.1%)	742
**2000**	2	1	1	159 (20%)	794
**2001**	3	2	1	169 (20%)	842
**2002**	5	3	2	157 (19%)	823
**2003**	2	2	0	127 (16.5%)	768
**2004**	3	1	2	157 (19.4%)	806
**2005**	3	2	1	166 (20.8%)	796
**2006**	4	2	2	141 (16.8%)	839
**2007**	4	3	1	153 (18.4%)	828
**2008**	4	2	2	146 (18%)	812
**Total**	71	43	28	3071(20.39%)	15056

**Table 2.** Malignant Melanoma Patients Based on Age Groups

Age Group	Number	%
**Under 40**	5	7
**40-49**	11	15.5
**50-59**	19	26.7
**60-69**	12	17
**70-79**	15	21.1
**80 and over**	9	12.7
**Total**	71	100

Table 3 shows the localization of the lesion and the treatment procedures. Face with 38% is the most common area of involvement. In total 60% of CMM lesions were in the head and face area. 53.5% of cases were treated with surgery, 12.7% with chemotherapy, 11.3% with radiotherapy and 22.5% with combination of radiation and chemotherapy. 

**Table 3.** Localization of Involved Area with CMM

Location of Lesion	Number	%	Treatment Type	Number	%
**Face**	27	38	Surgery	38	53.5
**Limbs**	19	26.7	Chemotherapy	9	12.7
**Head and Neck**	17	24	Radiotherapy	8	11.3
**Other body site**	8	11.3	Combination	16	22.5
**Total**	71	100	Total	71	100

Table 4 shows the mean five year survival rates and standard errors based on gender. The mean survival rates for women were better than men (80.5% and 76.3% respectively). Also table 4 shows that highest survival rate (based on career) belong to clerks with 92.1%, and for the treatment procedures correspond to surgery with 85.6%. Table 5 shows mean cumulative probability survival according to gender. 

**Table 4.** Mean Survival Time According to Gender, Occupation and Type of Treatment

Survival, 5 Years	Mean of Survival	Standard Error
**Sex**		
Male	76.3	0.010
Female	80.5	0.013
**Occupation**		
Farmer	79	0.010
House wife	73.2	0.018
Clerk	92.1	0.025
Worker	88.2	0.021
**Treatment Type**		
Surgery	85.6	0.010
Chemo-therapy	31.9	0.014
Radio-therapy	64.7	0.019
Combination therapy	43.2	0.025

**Table 5.** Cumulative Probability Survival According to Gender

	Mean			
	Estimate	Std. Error	95% Confidence Interval	
			Lower Bound	Upper Bound
**Male**	43.603	5.129	33.551	53.655
**Female**	37.202	4.333	28.709	45.695
**Overall**	44.648	3.988	36.831	52.464

## 4. Discussion

Epidemiological studies around the world indicate that CMM accounts for about five percent of all types of skin cancer leading to about three-quarters of all deaths due to skin cancers (18).

Table 6 shows incidence of CMM for 23 selected countries ( 19 ). The highest have been reported from Queensland, Australia with 56 new cases per year per 100,000 for men and 43 for women ( 20 ). This rate for the United States is 14 for men and 11.33 for women. In Northern Europe it is less than five per 100,000 ( 18 , 21 ). The highest rates belongs to Scandinavia (especially Sweden) with 15 cases per 100,000 inhabitants, and the lowest rates are from the Mediterranean countries (about 5 – 7 cases per 100,000 inhabitants per year) ( 22 ). 

**Table 6.** Incidence of Cutaneous Malignant Melanoma (per 100,000) for 23 Selected Countries

Country	Male		Female	
	Crude	ASR	Crude	ASR
**Australia**	51.6	40.5	40.7	31.8
**New Zealand**	45.2	36.7	44.4	34.9
**Sweden**	19.8	12.6	19.9	13.3
**U.S.A.**	16.4	13.3	12.9	9.4
**Denmark**	14.8	10.6	17.6	13.0
**Switzerland**	12.5	9.3	15.0	11.1
**The Netherlands**	12.2	9.4	16.7	12.9
**Austria**	11.5	8.8	15.4	10.4
**Canada**	10.6	8.2	10.6	8.0
**Hungary**	10.3	7.6	10.3	6.8
**Israel**	9.7	9.4	11.0	9.8
**Germany**	9.3	6.5	11.4	7.1
**France**	8.6	6.8	11.1	7.9
**U.K.**	8.3	6.1	11.3	7.7
**Poland**	6.6	5.6	8.6	6.7
**Italy**	6.5	4.6	8.2	5.5
**Russian Federation**	6.3	5.4	6.4	4.7
**Spain**	4.0	2.8	6.8	4.5
**South Africa**	3.8	6.4	3.6	4.8
**Brazil**	2.9	3.5	2.0	2.2
**Greece**	2.5	1.9	3.2	2.0
**Japan**	0.63	0.40	0.49	0.29
**China**	0.21	0.22	0.17	0.17
**Iran (Yazd)**	0.40	0.38	0.27	0.22

In Iran limited studies have been accomplished, with estimated incidence of 0.3 new cases of CMM per 100,000 per year (23). This study is consistent with the previous results indicating that the incidence of CMM is much lower compared to Non-Melanoma Skin Cancers (NMSCs). The incidence of CMM during this study (1987 – 2008) was relatively constant, which is in contrast to current literature that shows a rise in fair-skinned populations throughout the world for several decades (24, 25). Available data suggests that the frequency and distribution of CMM in men and women could be related to part of the body exposed to the sun (26). In an epidemiological study in Brazil, CMM affected men and women equally (5, 27-29), in Europe; a German study showed equal incidence in both sexes, whereas, in countries with a lower incidence, such as Great Britain, a higher incidence of CMM was found in women (30). In countries with a high CMM incidence, such as Australia, there was a higher rate of CMM in men (31-33).

A study in Iran – Isfahan (1988) showed that melanoma was 1.5 times more frequent in men than in women. (16). This study also showed that CMM is 1.5 times more frequent in male than female. It is very likely that clothing style and body covering routines, and the lower levels of employment of women in outdoor activities in this area, may play a role in such differences. Skin cancers mainly appear in the sixth, seventh and latter decades of life. But CMM mostly appears about 10 years earlier as the literature suggests (1, 4, 11, 34, 35). In the present study more than 42 percent (30 out of 71) of CMM cases were between 40 – 60 years of age that shows a 10 year earlier onset of CMM in comparison with other types of skin cancers, which is in concordance with the literature.

Literature suggests that the anatomical site of CMM varies according to gender. This might represent cultural differences in body coverage. In men most of the tumors are localized on the trunk, in women the more common site is lower extremity as an exposed area, based on the type of clothes used in different parts of the world, followed by head and neck and upper extremities, with nearly equivalence in both sexes (36-38). This study showed that localization of CMM in this area differed from other parts of the world. About 60% (42 out of 71) of all CMMs evolved in head and neck area. This confirmed the important role of exposure to sun in the development of CMM. Based on our study we may conclude that:

1. CMM in Yazd is a low-incidence skin tumor that shows a relatively fixed incidence in the last decades.

2. Higher incidences of CMM were found in sun-exposed areas especially for men.

3. Skin cancers and CMM incidence in Iran is lower than western countries, and it seems that it relates to geographical zone, genetic factors, skin type, society-related customs (including clothing styles and body coverings), and socio-cultural issues and habits.
